# Twenty-Five Years of PSP Toxicity in Galician (NW Spain) Bivalves: Spatial, Temporal, and Interspecific Variations

**DOI:** 10.3390/toxins14120837

**Published:** 2022-12-01

**Authors:** Juan Blanco, Ángeles Moroño, Fabiola Arévalo, Jorge Correa, Covadonga Salgado, Yolanda Pazos, Silvia Calvo, Araceli Escudeiro Rossignoli

**Affiliations:** 1 Centro de Investigacións Mariñas, Xunta de Galicia, Pedras de Corón, 36620 Vilanova de Arousa, Spain; 2 Instituto Tecnolóxico Para o Control de Medio Mariño de Galicia (INTECMAR), Xunta de Galicia, Peirao de Vilaxoán s/n, 36611 Vilagarcía de Arousa, Spain

**Keywords:** PSP toxicity, multidecadal, prevalence, incidence, variability, cycles, mollusks, toxin, HAB, harmful algal bloom

## Abstract

Twenty-five years of paralytic shellfish poisoning (PSP) toxicity in Galician bivalves have been studied. PSP was detected in 4785 out of 73,740 samples of the commercially important bivalve species analyzed from 1995 to 2020. Its general prevalence in the area was 6.5%. Only 1.6% of all samples tested were over the regulatory limit (incidence). The maximum level of PSP in the area, 40,800 µg STX 2HCl-eq kg^−1^, was recorded in raft mussels from Bueu (PON-II, Pontevedra) in December 2005. The highest maximum PSP values were found in mussels, which were mostly affected by *Gymnodinium catenatum*, but not those of prevalence and incidence which were recorded in clams, mostly affected by *Alexandrium*. Average levels in mussels were higher than in any other studied species. Spatially, in general, the prevalence, incidence, maximum, and average PSP toxicity during episodes tend to decrease from south to northeast, but some hot points with high levels can be identified. PCA analysis separates the southern rías, associated to *G. catenatum* blooms, from the middle and northern ones, associated to *Alexandrium* blooms. Along the year, two main peaks of the four variables are observed, the first one in late autumn–winter and the other in summer, the summer peak being much more important for the infaunal species than for raft mussels. In the seasonal pattern obtained by time series analysis of the average PSP toxicity, the autumn-winter peak was only maintained (and very reduced) in the southern rías, indicating that this peak is seasonally much less important than the summer peak. The observed seasonality is expected based on the timing of the blooms of the two PSP-producing phytoplankton groups present in the area. Over the 25 years of monitoring, large differences in PSP toxicity have been observed. Apart from some special years, an ascending trend in prevalence and incidence seems to be present from 2011 to 2020. No trend seems to exist during the same period for average or maximum toxicity.

## 1. Introduction

Paralytic shellfish poisoning is a syndrome caused by the ingestion of saxitoxin or its analogs. The intoxication symptoms typically start with a tingling sensation around the mouth, followed by numbness, paresthesia, and, in extreme cases, paralysis of the extremities and respiratory muscles, which can lead to respiratory arrest and death. The first known intoxications were reported by Captain Vancouver in 1791 after eating mussels from British Columbia. At the beginning of the past century, many cases were detected from both that area and others along the North American coast. After developing a mouse bioassay to detect this kind of toxicity [1,2,3], its presence in many other areas of the world was progressively detected and associated to human intoxications or fatalities.

PSP toxins are produced by several groups of algae, but, in marine environments, the main producers are dinoflagellates from the genus *Alexandrium* and from the species *Gymnodinium catenatum* and *Pyrodinium bahamense*. The toxins can accumulate in large amounts in bivalve mollusks (among other species) that produce intoxications when they are consumed. Due to the high risk this group of toxins poses to human health, its maximum allowable level in food has been generally established as 800 µg of STX 2HCl equivalents kg^−1^ of food, in many countries [4,5,6,7].

Galicia is the top bivalve (particularly mussels) producer in Europe, with more than 238,000 metric tons per year since 2003 [8]. Apart from mussels, other bivalve species are also harvested (more than 500 metric tons per year since 2003), increasing the economic and social importance of this food resource. Part of this production is exported, mostly to other European Countries.

In 1976, PSP was detected for the first time in Galicia when ten people became intoxicated after consuming mussels from the Ría de Arousa [9]. During the same outbreak 113 people were affected in Europe, due to the consumption of contaminated mussels exported from the area [10]. After this event, a monitoring program was established [11].

A precise knowledge of the interspecific, spatial, and temporal variations of the toxicity is very important to fine-tune the monitoring systems, and hence to reduce the risk for humans. The interspecific variation is known to be high. Different bivalves have different capabilities to accumulate PSP toxicity [12], mostly because they do not accumulate the toxins in the same way, but also because they can bio-transform the different analogs into others with higher or lower toxic potency, modifying the toxicity of the mixture without changing the total toxin amount [12,13,14,15,16,17,18,19,20,21,22,23,24]. The spatial variability is linked to the hydrography of the area, as shown by Anderson [25] for *Alexandrium*, and to the requirements of the causative species. The seasonal variation in the Northern Hemisphere is usually characterized by a high incidence in spring-summer months, associated to the development of *Alexandrium* populations [12,26,27], but in some areas, such as Portugal, where *Gymnodinium catenatum* is the main causative species, the yearly maximum usually takes place in autumn or winter [28]. Long term variability has also been studied on a few occasions, showing different patterns [12,28].

In Galicia (Spain), PSP toxicity in commercially important bivalves was monitored by Health Authorities from 1976 to 1995, when an institution belonging to Galician Fisheries Authorities, Intecmar, took charge of all controls needed to fulfill European Regulated hygiene rules for live bivalve mollusks between them those related to harmful algal blooms. Mouse bioassay was the method used to quantify PSP toxicity until January 2021, when a LC-FLD (liquid chromatography with fluorescence detection method) with pre-column oxidation of the toxins replaced bioassays, following regulation by the EU [29]. Since 1995, Intecmar has gathered a large dataset with more than 150,000 determinations, which allow to adequately describe the impact of PSP on the different bivalve species, the possible trends, and the spatial and seasonal variation in Galicia.

**Figure 1:**
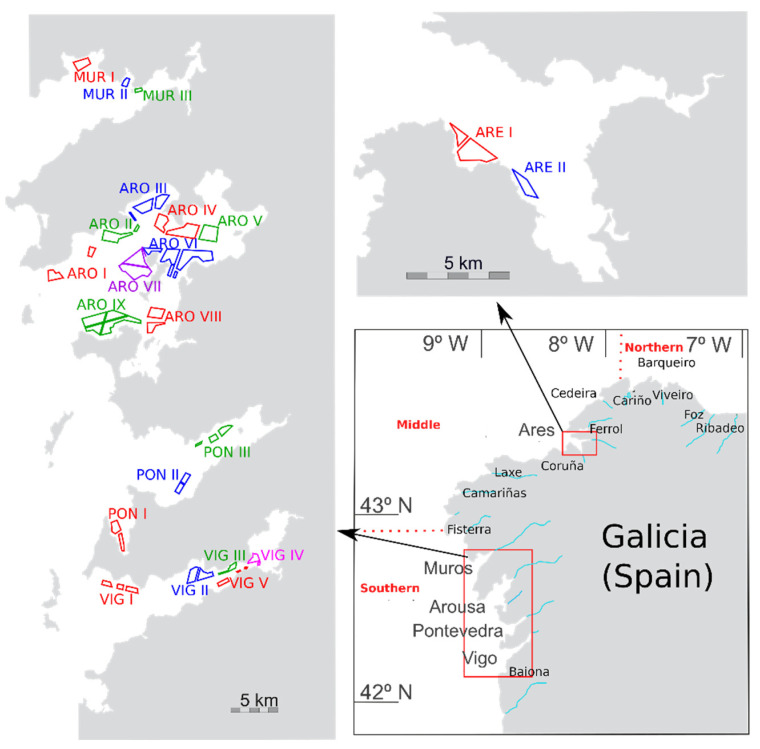
Area of study. Rías from which samples were taken (**lower right** panel), and mussel production areas (two other panels).

In this work, prevalence (proportion of the samples in which PSP was detected), incidence (proportion of the samples with PSP toxicities above 800 µg STX-eq. kg^−1^), and the main sources of variability of the PSP toxicity in bivalves of the area (Figure 1) have been characterized. This includes the apparent intoxication and detoxification rates of each species, the comparison of the levels in mussels (used in this case as the sentinel species) with other bivalves, and the analysis of the trends or cycles which might have taken place over a 25-year period.

## 2. Results

### 2.1. General

PSP toxicity was detected in 4785 out of 73,740 samples of the commercially important bivalve species analyzed from 1995 to 2020. The proportion of this number corresponding to each bivalve species studied is shown in Table S1. Its general prevalence in the area was 6.5%. Only 1.6% percent of all samples tested were over the regulatory limit. The maximum level of PSP toxicity in the area, 40,800 µg STX 2HCl-eq kg^−1^, was recorded in raft mussels from the culture area PON-II, in the ría of Pontevedra (PON) on 12 December 2005.

During PSP outbreaks, its mean concentration was 1191 µg STX 2HCl-eq kg^−1^ of mollusk meat, but only half of the observations (median) were above 530 µg STX 2HCl-eq kg^−1^. The top 5% concentrations were above 3842 µg STX 2HCl-eq kg^−1^ (5% quantile).

Prevalence was more associated to the presence (in integrated samples of the water column, when not obtained from the intertidal zone) of *Alexandrium* (384 detections) than to *G. catenatum* (276 detections), while incidence was more related to *G. catenatum* (217 vs. 102 for *Alexandrium*).

### 2.2. Interspecific Variation

**Figure 2:**
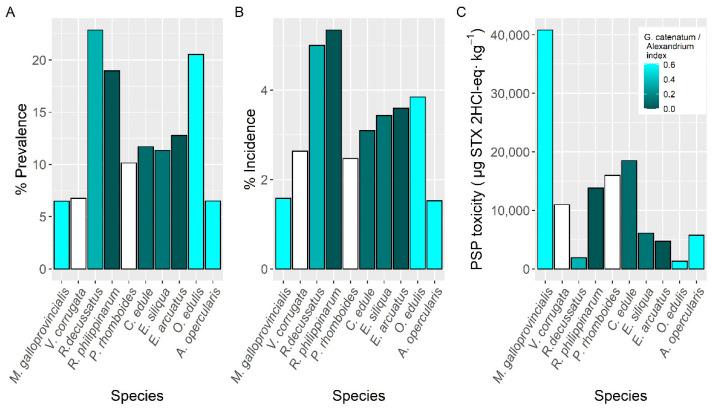
Proportion of the samples in which PSP toxicity was detected (Prevalence) (**A**), in which the concentration was above the regulatory limit (Incidence) (**B**), and maximum PSP toxicity attained by the main bivalve species (**C**). Colors indicate the proportion of *Gymnodinium catenatum* in relation to *Alexandrium* spp. White bars indicate that none of the phytoplankton species were found associated to PSP toxicity.

In all cases, the prevalence of PSP toxicity, which varied with the shellfish species studied, was below 25% (Figure 2). The highest prevalence (22.9%) was found in the clam *Ruditapes decussatus*, followed by European oyster *Ostrea edulis*, and *Ruditapes philippinarum* (20.5 and 19.0%, respectively). The minimum values were around 6% and corresponded to the mussel *Mytilus galloprovincialis*, the clam *Venerupis pullastra*, and the scallop *Aequipecten opercularis* (Figure 2). These observations, however, are biased because, in most cases, species other than mussels are not sampled until the detection of toxicity in the mussels used as sentinel.

The proportion of samples whose concentration was higher than the regulatory limit (incidence) was the lowest in the pectinid *A. opercularis*, which was mainly sampled in late autumn and winter. It ranged from 1.6% in mussels to 5.3% in *R. philippinarum*, and 5% in another clam, *R. decussatus*. All other studied species had incidences between 2.5% and 3.8% (Figure 2).

The maximum PSP toxicity levels in the area, 40,800 µg STX 2HCl-eq kg^−1^, were recorded in the raft mussel *M. galloprovincialis*, followed, with less than a half of its toxicity, by the cockle *C. edule* (18,500 µg STX 2HCl-eq kg^−1^) (Figure 2). The lowest maximum PSP toxicity per species (1280 µg STX 2HCl-eq kg^−1^) corresponded to the oyster *O. edulis* followed by the clam *R. decussatus* (1890 µg STX 2HCl-eq kg^−1^) (Figure 2).

**Figure 3:**
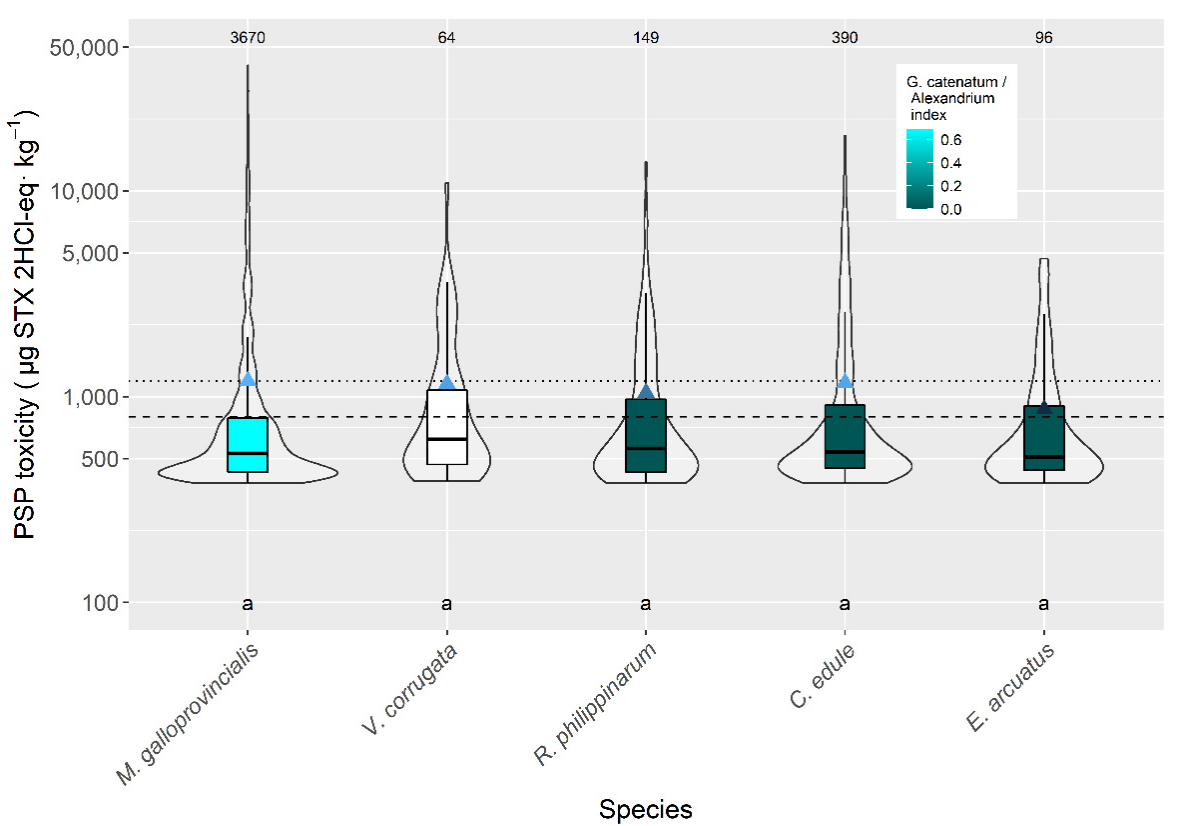
PSP toxicity during the toxic episodes in the main species of the area. Triangles = means, horizontal lines of the box = 25, 50, and 75% quantiles, extremes of the vertical lines from the box = range excluding outliers, dots = outliers. The outer shape (violin) represents the distribution of the data. The dashed line represents the regulatory threshold and the dotted one represents the average level in raft mussels. The figures at the top of the plot are the number of observations. The averages of the species sharing the letters at the bottom of the plot were not significantly different (Tukey HSD test). Colors indicate the proportion of *Gymnodinium catenatum* in relation to *Alexandrium* spp. White bars indicate that none of the phytoplankton species were found associated to PSP toxicity.

Between the five species with more than 50 observations available, the average toxicity during the PSP episodes was very similar and not statistically different. The highest level corresponded to *M. galloprovincialis*. (1193 µg STX 2HCl-eq kg^−1^) and the lowest to *Ensis arcuatus* (869 µg STX 2HCl-eq kg^−1^) (Figure 3).

Prevalence and incidence were higher in the shellfish species more affected by *Alexandrium* populations while maximum levels were attained in the species most affected by *G. catenatum.*

**Figure 4:**
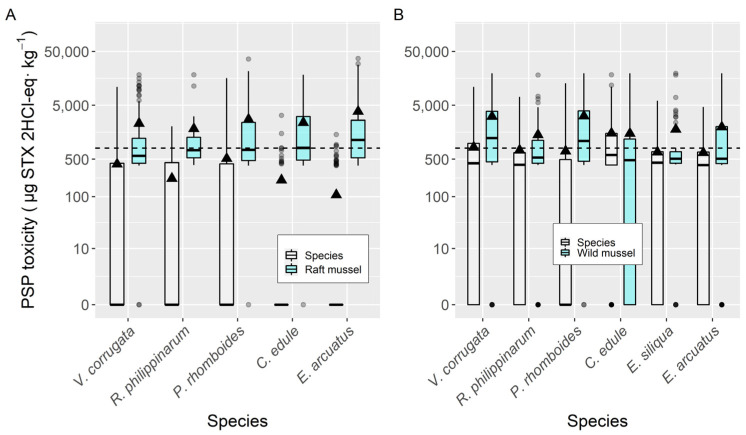
Comparison of PSP toxicity in raft mussels (**A**) and wild mussels (**B**) with other bivalve species, using data (maximum) collected from the same week and the same area. All symbols as in Figure 3.

All bivalve species had lower PSP toxicity than raft mussels when the data for raft mussels and other bivalve species that were collected from the same area and the same week were considered (Figure 4). When compared to wild mussels (also sampled from the same estuary in the same week), the average concentrations of all the analyzed bivalve species were also lower than those of mussels, with the only exception being the cockle *C. edule*, which had PSP toxicity levels slightly over those found in mussels (Figure 4). The highest differences recorded with raft and wild mussel corresponded to *E. arcuatus* and *Polititapes rhomboides*, respectively.

In all species that could be compared with wild mussels from the same week and production area, less than 22% of the samples tested PSP positive (over the regulatory level) when mussels were below that level (Figure S1).

### 2.3. Spatial Variation

**Figure 5:**
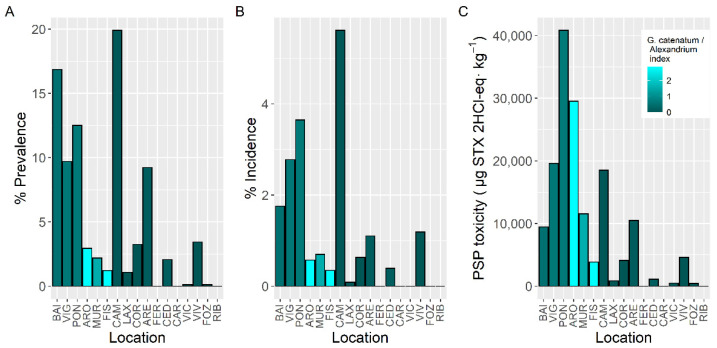
Proportion of the samples in which PSP toxicity was detected (Prevalence) (**A**), in which the concentration was above the regulatory limit (Incidence) (**B**), and maximum PSP toxicity attained by the bivalves in each estuary (**C**). Colors indicate the proportion of *Gymnodinium catenatum* in relation to *Alexandrium* spp. Locations are ordered from south (**left**) to northeast (**right**).

PSP toxicity prevalence showed, in general, a decreasing trend from the southernmost rías to the northeast extreme of the sampling area (Figure 5). There were, nevertheless, some noticeable exceptions, such as the ría of Camariñas (CAM, in which the maximum prevalence, 20%, was recorded), Ares (ARE), and Cedeira (CED), in the middle part of the Galician coast, and Viveiro (VIV), in the north. The minimal prevalence, with no PSP toxicity detection up to date, was recorded in Ferrol (FER), Cariño (CAR), and Ribadeo (RIB).

Incidence did not show exactly the same geographical pattern than prevalence (Figure 5). As in the case of prevalence, the highest value corresponded to Camariñas (CAM), but the second one did not correspond to the southernmost sampling location but to the ría of Pontevedra (PON). The minimum levels were recorded in the northernmost sampling locations, again with the exception of the rías of Viveiro (VIV) and Cedeira (CED).

Maximum PSP toxicities followed a similar pattern but with the rías of Pontevedra (PON) and Arousa (ARO) as the locations in which the maximum toxicities were detected. Camariñas (CAM) was, in this case, the fourth with most toxic values. The northernmost rías had, in general, low maximum toxicities, with the exception of Viveiro (VIV) (Figure 5).

Prevalence and incidence were, in general, higher in the rías more affected by *Alexandrium*, while maximum levels were recorded in rías affected by *G. catenatum.*

**Figure 6:**
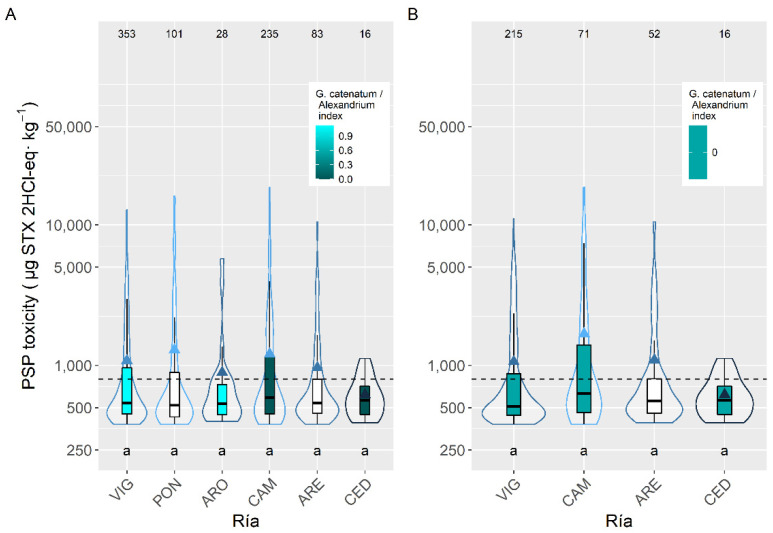
PSP toxicity in the main bivalve species with the exception of mussels (**A**), and in the cockle (**B**), during the toxic episodes in the Galician rías. All symbols as in Figure 3.

To study the levels of PSP toxicities during the episodes, mussels were initially excluded from the analysis to eliminate (i) the bias due to the presence or not of mussel rafts (mussel culture in rafts only exist in five rías, four of them in the southernmost area), and (ii) the fact that wild mussels are not usually sampled during the episodes, because in Galicia this species cannot usually be commercialized being mainly used as sentinel species. In this dataset, more than 10 PSP toxicity values above the detection limit of the method were detected only in six out of 17 rías studied when all bivalves except mussels were considered. This number became five when only cockle *C. edule*, which is the species more widely distributed along the rías, is used. The differences among those rías were, in general, relatively small (from 620.0 to 1289.5 µg STX 2HCl-eq kg^−1^) and non-significant statistically (Figure 6).

If the data corresponding to the cockle, which was the most sampled species after the mussel, are used, the observed pattern is very similar to that observed for mussels. The only noticeable differences are that no PSP episodes were detected in the Ría de Arousa, and that the average PSP toxicity in the Rías of Camariñas (CAM) and Ares (ARE) are higher than in the other rías.

In infaunal species the PSP toxicity average during the episodes did not increase with the incidence of *G. catenatum*. In fact, the maximum values were recorded in Camariñas (CAM), where *A.* cf. *minutum* is the only PSP-producing species detected.

When mussels (raft and wild) were included in the spatial variation analyses, PSP toxicity was detected above the limit of quantification (LOQ) in 12 out of the 17 rías sampled. The observed pattern was characterized by relatively high levels in the rías from Vigo (VIG) to Camariñas (CAM) and low levels in all others, with the exception of Ría de Viveiro (VIV) on the north coast (Figure S2).

**Figure 7:**
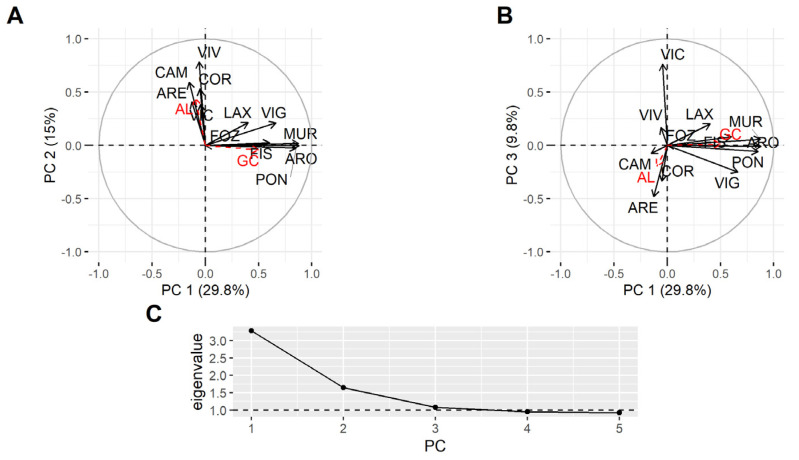
Loadings of the three principal components (**A**,**B**) and their corresponding eigenvalues (**C**). All data was included. AL and GC (in red) are the abundances of *Alexandrium* spp and *Gymnodinium catenatum*, and are supplementary variables which were not included in the analysis.

Principal component analysis (PCA), using all data, showed that not all rías behaved in the same way in relation to the PSP toxicity in bivalves (Figure 7). The first component shows that most of the PSP toxicity variation in the area was due to the southernmost rías (PON, VIG, ARO, MUR), and it was associated with the abundance of *G. catenatum*. The second component, which was associated with the abundance of *Alexandrium*, was determined by the middle rías with high loadings of VIV and CAM, whose variation was practically independent of that in the southern rías.

Taking into account that most mussel rafts are located in the southern rías, a new analysis excluding raft mussels was carried out. Unfortunately, in this case, most rías had to be excluded from the analysis (in this case, all southern rías but Vigo (VIG)) because wild mussels are not sampled in most areas in which there are mussel cultures. Additionally, the available data from rías without raft mussels were not well synchronized with other rías. The results obtained are consistent with the observation that the episodes of species other than mussels were mostly affected by *Alexandrium*, and not by *G. catenatum.* The first component was mainly determined by Viveiro and Ares and related to *Alexandrium*, and the second was defined by Camariñas and Cedeira (in opposite directions) and not related to any of the phytoplankton species (Figure S3).

**Figure 8:**
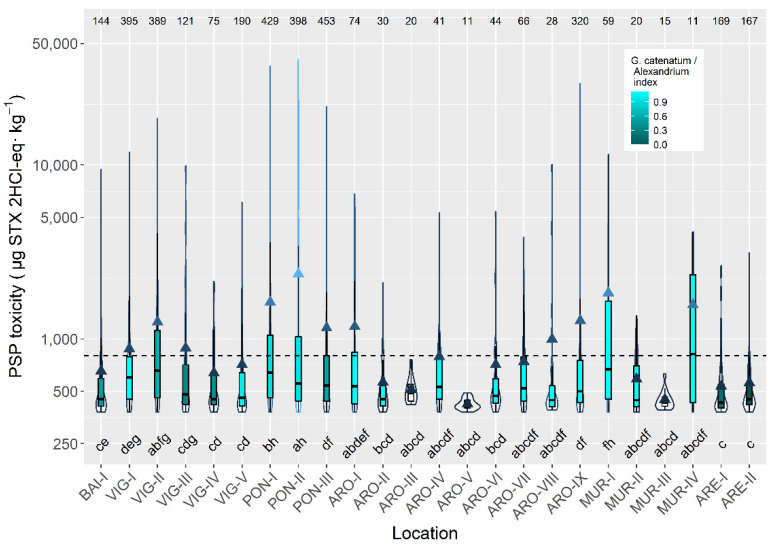
PSP toxicity in the main mussel production areas, during the toxic episodes in the Galici-an rías. All symbols as in Figure 3.

In raft-cultured mussels, the highest mean toxicity levels were recorded in two production areas of the rías of Pontevedra and Muros. The lowest values were found in the inner production areas of the Arousa and Muros, and in the whole ría of Ares (Figure 8).

### 2.4. Seasonality

**Figure 9:**
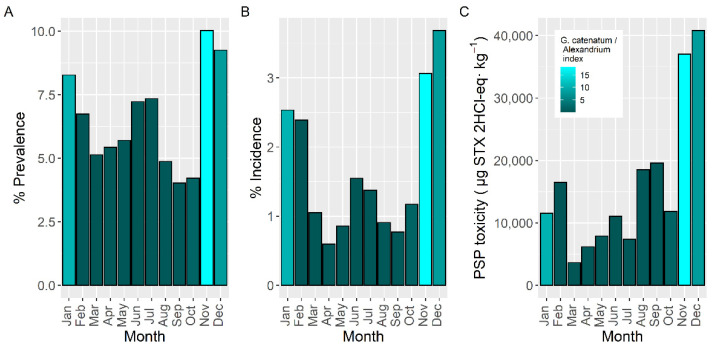
Proportion of the samples in which PSP toxicity was detected (Prevalence) (**A**), in which the concentration was above the regulatory limit (Incidence) (**B**), and maximum PSP toxicity attained by the bivalves in each month of the year (**C**). Colors indicate the proportion of *Gymnodinium catenatum* in relation to *Alexandrium* spp.

The prevalence and incidence of PSP toxicity presented two maxima during the year (Figure 9). The first took place in late autumn-winter (November to February), associated mostly with *G. catenatum*, and the second in summer (June–July), associated with *Alexandrium*. The maximum attained toxicities present an additional peak in September-October which was more important than that recorded in June–July. The minimal values of incidence and the lowest maximum toxicities attained took place in March–April.

**Figure 10:**
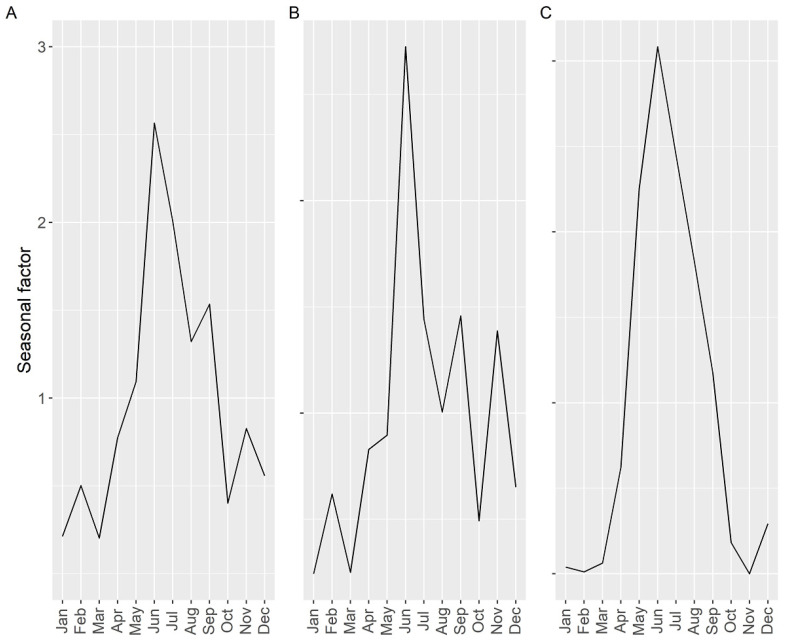
Seasonal pattern (ratio of actual value: deseasonalized value), of the average PSP toxicity, obtained by time series analysis for Galician Rías including all of them (**A**), only southern ones (**B**) or only middle ones (**C**).

Seasonality of the average PSP toxicity (including samples with zero concentration), estimated by time series analysis, showed one main peak, in June, and two small peaks in November and February, coinciding the first one with the typical timing of the proliferations of *A.* cf. *minutum* and the other two with those of *Gymnodinium catenatum* (Figure 10). The autumn-winter peaks were associated with the southern rías and disappeared in the middle rías. It was not possible to compute the seasonality in the northern rías because of the high number of samples below the detection limit or missing data.

**Figure 11:**
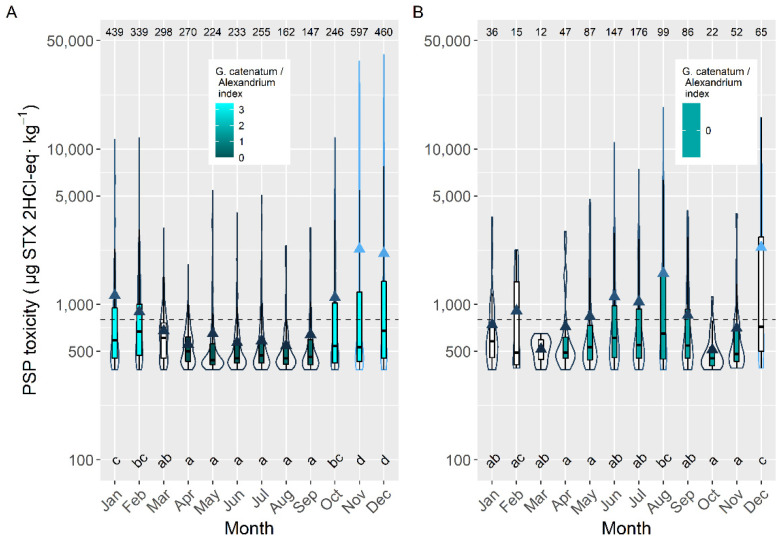
PSP toxicity over the year in raft mussels (**A**) and the other main bivalve species (**B**), during the toxic episodes in the Galician rías. All symbols as in Figure 3.

The intensity of the toxic episodes of PSP in raft-cultured mussels showed a concentration maximum in late autumn and early winter, November and December, descending progressively until April, and maintaining approximately its level until September, when it started to increase (Figure 11). Its dependence on *G. catenatum* blooms is evident. Peaks in June–July, August, or January-February, which could be expected from the data of prevalence, incidence and maximum level attained, were not present, showing the limited incidence of *Alexandrium* on raft-grown mussels. When mussels were excluded from the analysis, the seasonal pattern obtained was more consistent with the observations of prevalence, incidence, and maximum concentration, showing the expected peaks but with different importance. In this case, *Alexandrium* played an important role.

### 2.5. Time Course of the PSP Toxicity in Different Locations

**Figure 12:**
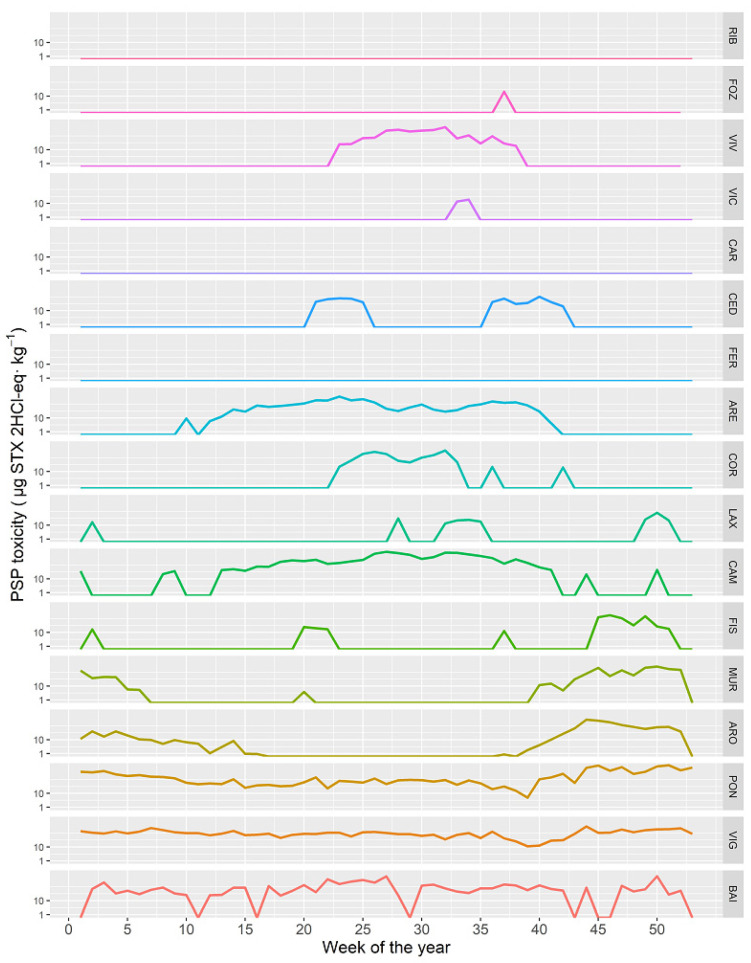
Average PSP toxicity along the year in the sampling locations along the Galician coast, from the southernmost ones (**lower** panels) to those in the extreme northeast (**upper** panels).

The time course of the PSP toxicity varied between the different Galician rías. In Baiona (BAI), Vigo (VIG), and Pontevedra (PON), the PSP toxicity levels did not vary substantially throughout the year (Figure 12). From there to Fisterra (FIS), the levels recorded from spring to autumn became progressively lower, coinciding with the usual geographical distribution of *G. catenatum.* To the north of this location, the autumn-winter toxicities practically disappeared, and the summer toxicities, after an initial high level in Camariñas (CAM), also decreased to the Northeast. These summer toxicities, notwithstanding, seem to have, apart for the commented general trend, an important local component, because PSP toxicity levels were low or even zero in some rías (Figure 12).

### 2.6. Interannual Differences

**Figure 13:**
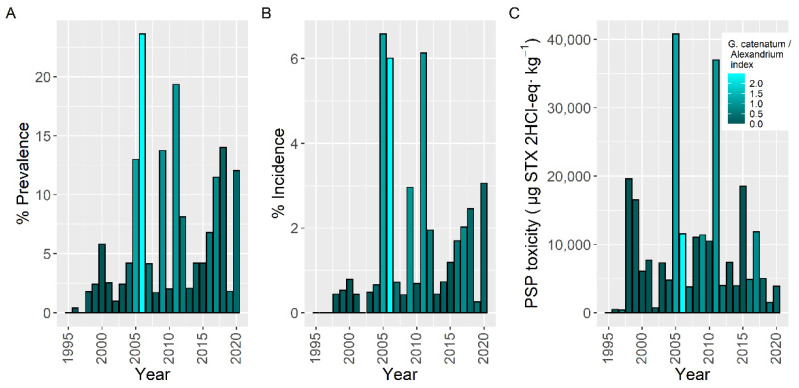
Proportion of the samples in which PSP toxicity was detected (Prevalence) (**A**), in which the concentration was above the regulatory threshold (Incidence) (**B**), and maximum PSP toxicity attained by the bivalves in each year (**C**). Colors indicate the proportion of *Gymnodinium catenatum* in relation to *Alexandrium* spp.

Variation of prevalence and incidence along the sampling period followed a similar pattern. Maximum levels attained, however, did not. From 1995 to 2004, both prevalence and incidence were relatively low. In 2005 and 2006 there was an important peak, especially of incidence. After that, there were two new peaks in 2009 and 2011–2012, and then, after a minimum in 2013, there was an increasing trend broken only in 2019. Maximum levels attained showed peaks in 2005 and 2011, roughly coinciding with prevalence and incidence, but had relatively higher values before 2004, and showed a decreasing trend since 2015 (Figure 13).

**Figure 14:**
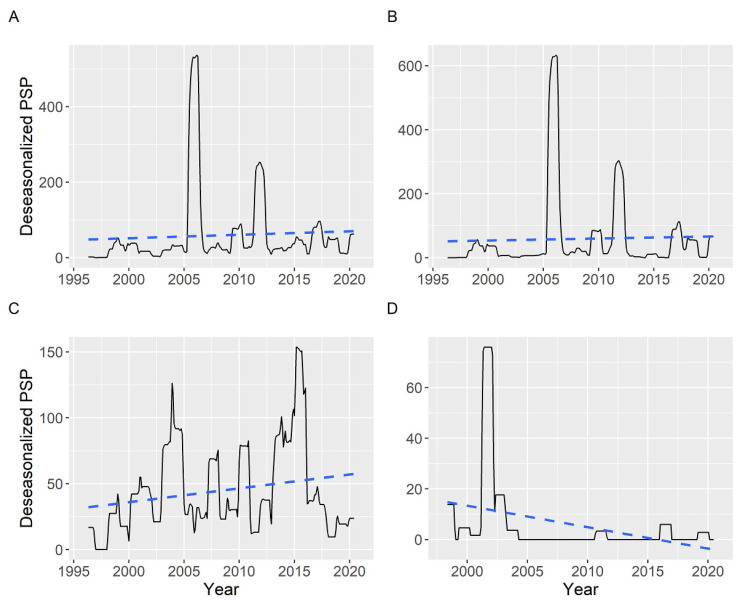
Deseasonalized average PSP toxicity corresponding to all (**A**), southern (**B**), middle (**C**), and northern rías (**D**). The dashed lines are the linear trends fitted by regression.

Once deseasonalized by time series analysis, no trend was detected when the average PSP toxicities (including zeros) corresponding to all of the southern rías were examined (Figure 14). The middle rías seem to have a slight increasing trend and the northern rías a decreasing trend, but this was based on a small data set.

The partial autocorrelation of the time series (including zeros), when all data or the data of the southern rías were analyzed, showed two main periodicities: 5.9 and 6.1 years, with positive and negative autocorrelations, respectively. In the middle and northern rías, the periodicity seems to be different, with positive autocorrelations at 1 and 3.2 years (not statistically significant in the case of the northern rías) (Figure S4).

**Figure 15:**
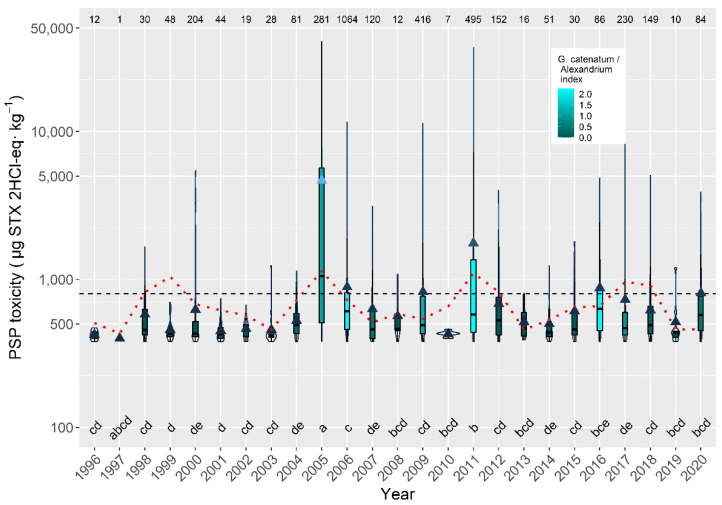
PSP toxicity in the episodes along the sampling period in raft mussels. The dotted line is the result of fitting the combination of three sinusoids with periods of 3.2 and 5.9 years. All other symbols as in Figure 3.

The annual average toxicity per episode in raft-cultured mussels over the sampled period usually varied between approximately 300 and 900 µg STX 2HCl-eq kg^−1^. There were, notwithstanding, two exceptions, corresponding to 2005 and 2011, in which the average levels attained approximately 5000 and 2000 µg STX 2HCl-eq kg^−1^, respectively. In those two years, intense *G. catenatum* blooms were recorded. In general, the periodicity observed for the average concentration also seems to apply for the magnitude of the episodes (Figure 15).

### 2.7. Apparent Intoxication and Detoxification

The apparent intoxication and detoxification rates were computed from the change in PSP toxicity recorded in bivalves between two consecutive weeks. The true rates could not be computed because, while mollusks are increasing their toxins content, depuration is simultaneously occurring, and consequently the observed increase is in fact the result of the balance between the absorption and true depuration. The opposite happens during the depuration period, in which the depurated toxin would be underestimated because some toxic cells could still be ingested by the bivalves. With this approach, therefore, only apparent rates, and not true ones, can be computed.

**Figure 16:**
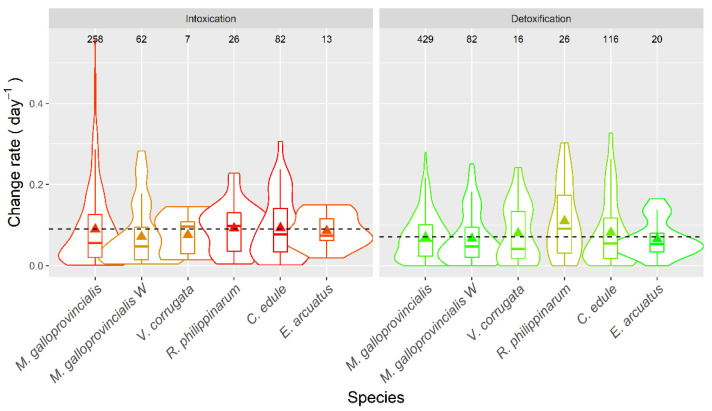
PSP apparent intoxication and detoxification rates in the main bivalve species of the Galician rías. Raft and wild mussels (W) were analyzed separately. The dashed line represents the levels corresponding to the raft mussels. The numbers in the upper part of the figure are the number of observations. All other symbols as in Figure 3.

Average apparent intoxication rates were, in general, low (Figure 16), with averages around 0.1 day^−1^, which means a doubling of PSP toxicity in the bivalves would take, on average, ~7 days. With the highest intoxication rates recorded, notwithstanding, this time would be reduced to slightly more than one day. The apparent depuration in all species but the manila clam *R. philippinarum*, was slow, with averages between 0.065 and 0.082 day^−1^ and rarely reaching 0.5 day ^−1^. The apparent depuration observed in *R. philippinarum*, 0.11 day^−1^, was faster than the recorded in the other bivalve species studied.

All species showed apparent intoxication and depuration rates that were similar to those of the raft mussel. Only the depuration rate of *R. philippinarum* was noticeably higher than that of raft mussel (*C. edule* and *V. corrugata* had slightly higher depuration rates).

## 3. Discussion

### 3.1. General

PSP toxicity detection in bivalves from Galicia was recurrent but not frequent while measured by mouse bioassay. This toxicity was only found in 6.5% of the samples obtained by the monitoring system from 1995 to 2020. Similar levels have been found in the Bay of Plenty, which is located in an area of New Zealand considered to have a chronic problem of PSP toxicity [30]. In Portugal, the equivalent prevalence between 2015 and January 2022 (computed from the data obtained by IPMA [31] by the HPLC-FLD reference method) was 2.2%, much lower than in Galicia. The data obtained by Intecmar during 2021 using the HPLC-FLD reference method of the European Union [32] suggest that the presence of this toxicity could be approximately 12 times more frequent. Consequently, during the studied period, it would have been detected in approximately 80% of the samples. In Portugal, PSP toxicity prevalence was 42.7%, nearly a half of that recorded in Galicia. This is consistent with the fact that in Portugal there are only PSP episodes originated by *G. catenatum*, which blooms mainly in late autumn, while in Galicia episodes of both *G. catenatum* and *A.* cf. *minutum*, which blooms mainly in summer, take place.

The incidence of PSP toxicity on bivalve populations was much smaller than its prevalence. The estimated toxicities were above the regulatory limit in only 1.6% of the analyzed samples. The incidence is, therefore, similar to that found for domoic acid (ASP) from the same area (1.3%) [33] but substantially lower than for lipophilic toxins, which was ~10.3% (estimated using a shorter period of time) [34]. The estimated incidence in Portugal (1%), like prevalence, was smaller than in Galicia. It was, however, higher proportionally to prevalence, probably because *G. catenatum* is more toxic than *A.* cf. *minutum* in the area [35,36,37]. In Great Britain, the recorded incidence seems to be even lower than in Portugal, with less than 0.3% of the analyzed samples being over the regulatory limit [38].

The maximum PSP toxicity, 40,800 µg STX 2HCl-eq kg^−1^, was recorded in *M. galloprovincialis*. This concentration is substantially lower than others recorded in different geographical locations, which seems to indicate that, in Galicia, this kind of toxicity entails a much lower risk for human health, than in other countries or regions. In Chile, for example, the mussel *Aulacomya ater* attained 1,132,590 µg STX 2HCl-eq kg^−1^ [39], in Argentina, *M. edulis* attained 296,068 µg STX 2HCl-eq kg^−1^ [40], and in Portugal [41], Morocco [42], and Puget Sound (USA) [43], levels of 60,000 µg STX 2HCl-eq kg^−1^ have been recorded. In some other European areas, such as Scotland (43,000 µg STX 2HCl-eq kg^−1^) and Sweden (36,000 µg STX 2HCl-eq kg^−1^) [41], the maximum recorded levels are similar to the ones in this study. 

The maximum toxicity levels seem to be associated to *G. catenatum* blooms. This could be expected because, even when the cell concentration in the recorded blooms was 35 times higher for *Alexandrium*, the toxicity of *G. catenatum* in the area is much higher (nearly 90×) [35,36,37], and additionally because, in some blooms, only a part of the *Alexandrium* cell counts correspond to *A.* cf. *minutum*.

### 3.2. Interspecific Variation

Prevalence varied with bivalve species in general, with higher values in species more affected by *Alexandrium*, such as clams, than in those more affected by *G. catenatum*, such as mussels. As there were only minor differences in apparent intoxication and depuration rate, the persistence of the toxins in the bivalves does not seem to be the main reason for the observed differences in prevalence, pointing to the exposure to the toxic phytoplankton species (depending on the location of the cultures and natural beds) as the main cause. *Alexandrium* blooms are widely distributed along the whole Galician coast, being usually associated to local cyst germination and to areas of river run-off, especially affecting the inner parts of some rías, where most beds of infaunal species are located. The location of *G. catenatum* blooms, contrarily, are associated to offshore water masses, and in Galicia exclusively affect the southernmost part of the coast (up to Fisterra), where most mussel cultures are located. High cell abundances of this species are usually found in the most external parts of the rías [44].

Incidence had a pattern very similar to prevalence. Maximum toxicities, however, were attained in mussels, followed by cockles and clams. The same order has been found in Portugal [45], where mussels are not cultured in rafts and are nearly exclusively affected by *G. catenatum*, pointing to the depuration rate as the main cause of the observed differences. In this study, the apparent depuration rates of mussel, cockles and clams were inversely related to their maximum attained toxicities. Experimentally, Artigas et al. [46] showed that the true depuration rate is faster for cockles than for mussels.

The average magnitude of the episodes did not significantly vary with bivalve species. Notwithstanding, mussels and cockles had the highest toxicities during the episodes, while the scallop *A. opercularis* had the smallest. The three clams had intermediate values. When direct comparisons (same area same week) between mussels and other species are made, average toxicities in mussels are always higher that in the other bivalves, justifying its use as indicator species.

### 3.3. Spatial Variation

The geographical pattern of PSP toxicity prevalence and incidence seems to be strongly related to two different processes. One is the concurrent presence of the two toxigenic species in the southernmost rías, and the other seems to be the recurrent *A.* cf. *minutum* blooms in rías that can be considered hotspots, as their sediments were found to contain a high number of cysts of *A.* cf. *minutum* more than twenty years ago [47].

The PCA analysis shows the association of the rías to the south and to the north of cape Fisterra with *G. catenatum* and *Alexandrium*, respectively.

Regarding the production areas where mussel is cultured in rafts, the lowest average PSP toxicities were clearly linked to the northern location of the ría (Ares), and to the distance to the mouth of the ría. The more inside the ría the production area is located, the lower its average PSP toxicity (e.g., ARO-V and MUR-III). The opposite happens with the maximum levels, which were recorded in the outer part of the southern rías (e.g., PON-I, MUR-I and ARO-IX). This pattern also reflects the influence of *Alexandrium* and *G. catenatum*, whose blooms develop in the inner part of the rías, in the first case, and offshore, in the second [44].

### 3.4. Seasonality and Timing of the Episodes

As could be expected, the seasonality of the toxic episodes was clearly linked to that of the PSP-producer species [44,48]. For prevalence and incidence, a peak in late-autumn-early winter associated to *G. catenatum* and another one in late spring-early-summer, associated to *Alexandrium*, are observed. For maximum levels, an additional peak in August-September was present. This peak seems to be due mainly to *Alexandrium* because the maximum cell concentrations of this species were attained at these months (Figure S5), especially in the rías with the highest levels (Figure S6). In the southern rías, nevertheless, *Gymnodinium catenatum* could probably have contributed to this early autumn peak because its blooms begun to appear during this time, and a mixture of species was observed (Figures S5 and S6). The seasonal pattern observed in Portugal [28,45] (and obtained from the data of IPMA monitoring since 2015 [31]) coincides with the one in Galicia, mainly in the late autumn peaks caused by *G. catenatum*, which is the main producer species in that country. In Portugal, the episodes start earlier (October). An early summer peak, much smaller than in Galicia, can also be observed that probably corresponds to *A. minutum*, which is also present in the area but rarely generates blooms [45] (obtained from the data of IPMA monitoring since 2015 [31]). A seasonal pattern similar to that of Galicia seems to also apply to the Atlantic coast of Morocco, where, in some years, PSP episodes attributed to *G. catenatum* took place in November, while other episodes attributed to *A. minutum* took place in spring–summer [42,49]. On the Mediterranean coast of Morocco, however, PSP episodes associated to the former species appeared almost at the beginning of the year (February–March) [50]. In other locations of the European Atlantic coast, in which the main PSP producers are *Alexandrium* species, such as the UK [38,51], Ireland [52], or France [53,54], the seasonality is characterized by a maximum in spring–summer coinciding with the component of seasonality attributed to *A.* cf. *minutum* in Galicia. In the Bay of Biscay (Spain and France), late autumn PSP toxicity outbreaks can appear, most likely associated to *A. ostenfeldii* [55,56].

Raft mussels had a different seasonal pattern than other bivalve species (mainly infaunal). PSP toxicity in mussels peaked in late autumn–early winter and there is not any relevant spring-summer maximum, indicating that it was mostly affected by *G. catenatum*. The other bivalves, in addition, present a spring-summer peak, originated by *Alexandrium*. Most likely, this difference is due to the locations that raft mussels and other bivalves occupy in the rías. Mussel rafts are mostly located in areas that avoid freshwater runoff, while other bivalves usually grow in sandy areas frequently affected by rivers. This difference could be expected from the characteristics of the PSP-producing species in the area. *Gymnodinium catenatum* blooms develop offshore and are advected to the rías, mainly to the outer part [57], while *A.* cf. *minutum* blooms develop mainly in the inner part of the rías and linked to freshwater [48,54,58].

When the seasonality was studied by time series analysis, the autumn–winter peak disappeared from the northern rías and was substantially reduced in the southern ones, while the spring–summer peak was clearly defined. This was due to the substantially different presence of *Alexandrium* and *G. catenatum* along the sampling period. *Alexandrium* produces PSP toxicity outbreaks every year, while *G. catenatum* can be absent from the area for years, thus reducing its effect on the seasonal component in the time series analysis.

The timing of the episodes in the different rías is clearly conditioned by two factors: (i) location, which conditions the influence of *G. catenatum*; and (ii) presence of *A.* cf. *minutum*, which is dependent on each particular ría. As in the southernmost ones where *G. catenatum* blooms exist, so in the rías in which *Alexandrium* also develops (Baiona, Vigo and Pontevedra), the spring-summer and the autumn–winter episodes overlay each other, and outbreaks can take place at any time in the year. In the three other rías affected by *G. catenatum* (Arousa, Muros, and Fisterra), *Alexandrium* seems to have no or little effect, and consequently the blooms start in autumn. In the remaining rías in which PSP toxicity outbreaks have been recorded, blooms start in spring.

### 3.5. Interannual Variability

The interannual variability of PSP toxicity in bivalves was high. The coefficient of variation (CV) of the mean toxicity, for example, was 150%. That variability, however, seems to have two components, one relatively stable (CV = 85%), due to spring-summer outbreaks, associated to *Alexandrium* blooms, and another one due to autumn–winter episodes, associated to *G. catenatum* blooms, which is nearly three times more variable (CV = 233%). The general variability therefore seems to be mostly determined by the autumn-winter toxicities, and consequently by *G. catenatum* blooms.

No clear trend has been observed along the studied period. In the southernmost rías, the relatively infrequent summer episodes make it impossible to find a trend. In the northernmost rías all episodes are infrequent with the same result. In the middle rías, even when a statistically significant trend was found, it seems clear that it is conditioned by some years with high average levels around 2015 which did not persist after 2017. There is, therefore, no clear evidence of a real trend in PSP toxicity. In Portugal, as in Galicia, PSP toxicity outbreaks due to *G. catenatum* are very heterogeneous in time, with some years with high incidence and high toxicity levels, followed by relatively long period without any significant effect of this species [28,45]. In Australia, the impact of that species seems to be more recurrent, even when high differences in PSP toxicity are recorded [59]. Other areas in which *Alexandrium* species are the main PSP producers seem to have a more recurrent pattern, as seen with spring-summer episodes in Galicia [12,38,60,61].

Some cyclic behavior in average PSP toxicity was also detected, but the results obtained are not conclusive. In the southern rías, the most affected by *G. catenatum*, significant partial autocorrelations of 5.9 (positive), 6.1, and 11.5 (negative) were detected. In the middle rías, most affected by *Alexandrium*, only autocorrelations of less than 1 year and of 3.2 years were significant. Vale [62] has suggested that solar activity can impact PSP episodes in the Portuguese and Galician coasts, and an 11-year cycle was found in solar activity and climate [63]. However, the autocorrelation is negative, and, therefore, a direct link of solar activity with the cycle observed in this study seems unlikely. There is a 3–5 year Chl *a* cycle in the northeast Atlantic [64], but no clear relationship could be established with PSP toxicities. 

The years with the highest PSP toxicity in Galicia (2005 and 2011) were also those with the highest domoic acid concentrations [33], which suggest that the two kinds of episodes can be linked to the same oceanographic processes, even when the producer organisms are substantially different.

### 3.6. Intoxication and Detoxification Rate

Apparent intoxication rates varied only slightly with the studied species. Wild mussels and *V. corrugata* had rates lower than those in raft mussels. The difference between mussels suggests that there should be an important component of localization, because, as the same species, their physiology is expected to be the same. The environment where wild mussels live is expected to have a more important amount of inorganic particles which could reduce the absorption efficiency of the toxins [65]. In Portugal, a lower intoxication rate was also found for the clam *Venerupis* (probably *V. corrugata*) than for *M. galloprovincialis*, but not for the cockle *C. edule* was found to have a similar rate [45,66].

The observed detoxification rates were also very similar for most species. Only the manila clam *R. philippinarum* depurated the toxins noticeably faster than the other studied species. Samsur et al. had already found high depuration rates for this species when intoxicated with both *G. catenatum* (0.15 day^−1^) [67] and with *Alexandrium catenella* [68] (recomputed for depuration discarding the first 48 h, when a substantial loss of non-absorbed toxins takes place). The observed rate for *M. galloprovincialis* fit well with published information such as [69] and references therein, and with those for different toxins of the PSP group [70]. A slightly faster depuration in the cockle *C. edule* than in mussels was also found in Portugal after a *G. catenatum* bloom [18].

## 4. Conclusions

Both the prevalence and incidence of PSP toxicity in the area are high. The maximum toxicity detected was 50 times the regulatory limit. It was substantially lower than the levels recorded in other geographical areas, such as Maine, Argentina, or Chile, but similar to those in Portugal, Morocco, and Scotland.

Two kinds of toxicity episode were observed. One takes place in Autumn–Winter, originated by *Gymnodinium catenatum* and relatively infrequent but intense. These episodes affected mostly raft mussels in the rías south of Cape Fisterra. The second type were originated by *Alexandrium* and were much more recurrent but less intense than those of the first type. They take place in Spring–Summer, especially in locations with a previous history of PSP toxicity (and in many cases, of *Alexandrium* cysts), being the only factor responsible for this toxicity in rías north of Cape Fisterra. In this kind of episode, mainly the infaunal bivalve species are affected.

No clear trend along the 25-year period could be detected, but 3.2- and 5.9-year cycles seem to exist.

Mussel is the most affected species and its average toxicities are always higher than those in the other monitored bivalves, justifying its use as sentinel species.

## 5. Materials and Methods

### 5.1. Sampling

Samples were collected with a minimum weekly frequency for all the production areas of Galicia in which harvesting of bivalve mollusks is allowed (depending on the exploitation plans). The mussel *Mytilus galloprovincialis* (culture in rafts or wild) was used as a sentinel species. When a toxic episode was detected in mussels, other harvested species (cockles, clams, oysters, razor-clams, and queen scallops) were sampled and analyzed. The harvesting of wild mussels for human consumption is not usually permitted in Galicia (except in restricted production areas). In some locations, mussel is used as a sentinel for infaunal species, and once the onset of a toxic episode was detected, the infaunal species were analyzed and wild mussels were not newly analyzed until that PSP episode ended.

Not all bivalve species were equally sampled, and some species have not been sampled throughout the whole year (Table S2). Raft mussels were subjected to the most intense monitoring, in part because they are used as sentinel and in part because the culture areas are divided into many sub-areas which are sampled individually. The percentages of samples corresponding to each species, and to each species in each month, are given in Tables S1 and S2, respectively.

*Gymnodinium catenatum* and *Alexandrium* samples were obtained from the locations in Figure S7 for the rías with raft cultured mussels by means of a 15 m garden hose and were obtained manually from the surface water at the same place as the bivalves in all other locations. Cell counts were carried out by the Utermöhl method. *Alexandrium* were only identified to the genus level in the counts because of the difficulty of discriminating species in settled samples, under the inverted microscope. Notwithstanding, in Galicia, *A.* cf. *minutum* is the only species in the genus associated to PSP events. When the sampling locations for phytoplankton were not the same as for bivalves, the cell abundance of the location nearest to the bivalves was used.

### 5.2. Reagents and Reference Solutions

Due to the long sampling period, different reagents were supplied by different vendors, but purity grade was maintained. Reagents: Hydrochloric acid (37%) ACS reagent grade, NaOH reagent grade, Milli-RO, or Elix water (Millipore Ibérica, Madrid, Spain). FDA STX Dihydrochloride Solutions were obtained from National Institute of Standards & Technology, Department of Commerce (Gaithersburg, MD, USA). Lugol’s solution and formaldehyde were obtained from different sources.

### 5.3. Toxin Extraction

Bivalve mollusks were opened, and the shells discarded. Soft tissues were pooled and homogenized with a blade blender. A 100-g aliquot of the homogenate was mixed with 100 mL of HCl 0.1 N, and the pH adjusted to 3 (range 2.5 to 3.5). The mixture was boiled for 5 min and cooled to room temperature, adjusting both the volume lost by evaporation and the pH (between 2 and 4) if necessary. To clarify the solution, it was allowed to decant or, if the solution was not clear enough, an aliquot was centrifuged for 5 min at 4000× *g*.

### 5.4. PSP Toxicity Quantification

On the basis of AOAC Official Method 959.08, a 1-mL aliquot of the obtained extract was intraperitoneally injected into each of three albino mice weighing 20 ± 1 g (with a few exceptions in the range 17–23 g), and the time to death was recorded. The toxicity was then computed by applying the Sommer table and a weight correction factor. The AOAC Official Method 959.08 was accredited at Intecmar under norm UNE-EN ISO/IEC 17025 since 1999 until 2021.

### 5.5. Data Processing

Data were processed in two ways: one to monitor the presence of PSP toxicity in the bivalves and the other to characterize the toxic episodes. In the first case, all samples were used. In the second, only those samples which contained detectable levels of PSP toxicity were used.

#### 5.5.1. Monitoring PSP Toxicity in Bivalves

With this aim, prevalence (proportion of the analyzed samples in which PSP toxicity was detected), incidence (proportion of the samples with PSP toxicities above 800 µg STX 2HCl-eq kg^−1^), principal component (to explore the spatial variability), and time series analysis (to explore the temporal variability, including trend, seasonal variation, and autocorrelation) were used.

Principal component analyses were carried out using the logarithmically transformed data (log(PSP toxicity + 1)), to avoid losing the data with zero concentration, utilizing the R packages FactoMineR [71] and factoextra [72].

Time series analysis was carried on all the available data. The R packages stat [73], forecast [74], and tseries [75] were used for the analysis and a multiplicative model was used for the decomposition.

#### 5.5.2. Characteristics of the Toxic Episodes

The characteristics of the episodes were described by statistics (mean, median, quartiles, range, and shape of the frequency distribution) using a combination of violin and boxplots. The R-package ggplot2 [76].

The *G. catenatum*/*Alexandrium* Index was computed using the concentrations of the species in plankton, by the following formula:

Index = log(*G. catenatum* + 1)/log(*Alexandrium* spp + 1).

## Data Availability

The data used for this study are available from Intecmar upon request.

## Supplementary Materials

The following are available online at https://www.mdpi.com/article/10.3390/toxins14120837/s1, Table S1: Percentage of the total number of samples analyzed corresponding to each bivalve species. Table S2: Percentage of samples of each bivalve species obtained each month; Figure S1: Differences of PSP toxicity between the main bivalves and wild mussels sampled from the same area at the same week; Figure S2: PSP toxicity in the main bivalve species during the toxic episodes; Figure S3: Loadings of the four principal components and their corresponding eigenvalues after excluding raft mussels from the analysis; Figure S4: Partial autocorrelation plots when all bivalve species are considered; Figure S5: Average concentration per month and percentage of the samples in which the species was present for *Alexandrium* and *Gymnodinium catenatum*; Figure S6: Maximum PSP toxicities per month in the rías with the highest PSP toxicity levels; Figure S7: Phytoplankton sampling stations in the five Galician rías where raft mussels are grown.
