# Supplementary material for: Twenty-Five Years of PSP Toxicity in Galician (NW Spain) Bivalves: Spatial, Temporal, and Interspecific Variations
Source: Toxins (Basel). 2022 Dec 1;14(12):837. doi: 10.3390/toxins14120837 (PMC9786697; doi:10.3390/toxins14120837)
Supplement: Supplementary file 1 [file toxins-14-00837-s001.zip › toxins-2024297-supplementary.pdf]

toxins

MDPI

Supplementary Materials: Twenty-Five Years of PSP Toxicity in Galician (NW Spain) Bivalves: Spatial, Temporal, and Interspecific Variations

Juan Blanco, Ángeles Moroño, Fabiola Arévalo, Jorge Correa, Covadonga Salgado, Yolanda Pazos, Silvia Calvo and Araceli Escudeiro Rossignoli

Table S1. Percentage of the total number of samples analyzed corresponding to each bivalve species.

Species	Samples	%
M. galloprovincialis Raft	56629	76.8
M. galloprovincialis Wild	10044	13.6
C. edule	3333	4.5
V. corrugata	949	1.3
R. philippinarum	786	1.1
E. arcuatus	752	1.0
P. rhomboides	365	0.5
E. siliqua	379	0.5
A. opercularis	262	0.4
R.decussatus	140	0.2
O. edulis	78	0.1
M. gigas	23	0.0

Table S2. Percentage of samples of each bivalve species obtained each month

	Month											
Species	Jan	Feb	Mar	May	Apr	Jun	Jul	Aug	Sep	Oct	Nov	Dec
M. galloprovincialis Raft	8	7.6	8.9	8.5	8.1	8.1	8.5	8.2	8.5	8.6	8.9	7.9
M. galloprovincialis Wild	9.8	9.4	10.3	8.8	7.6	7.2	7	7.2	7.9	8.1	8.2	8.4
V. corrugata	7.6	5.6	6.5	6.6	5.9	7.6	10	8.7	11	12.9	9.3	8.3
R.decussatus	1.4	2.1	2.1	7.1	10.7	19.3	10	12.9	10.7	8.6	3.6	11.4
R. philippinarum	2.5	1.8	2.3	6.6	11.1	13.6	13.9	13.2	11.1	9.8	8.5	5.6
P. rhomboides	3	0.5	3	7.4	9.9	6.6	14	11	12.6	14.5	10.1	7.4
C. edule	2.9	2.2	2.9	7.4	10.2	12.1	12	11.7	13	11.9	8.6	5.1
E. siliqua	0.3	1.1	2.4	3.2	13.2	15	19.5	22.7	14.5	4.7	2.6	0.8
E. arcuatus	1.3	0.4	1.5	5.6	12.8	14.8	16.6	9.2	16.2	10.8	5.9	5.1
M. gigas	0	0	0	0	13	13	17.4	21.7	26.1	8.7	0	0
O. edulis	7.7	6.4	2.6	3.8	14.1	1.3	3.8	3.8	5.1	5.1	38.5	7.7
A. opercularis	9.2	5.7	5.7	5	6.1	1.1	1.9	3.8	6.5	19.1	24.8	11.1

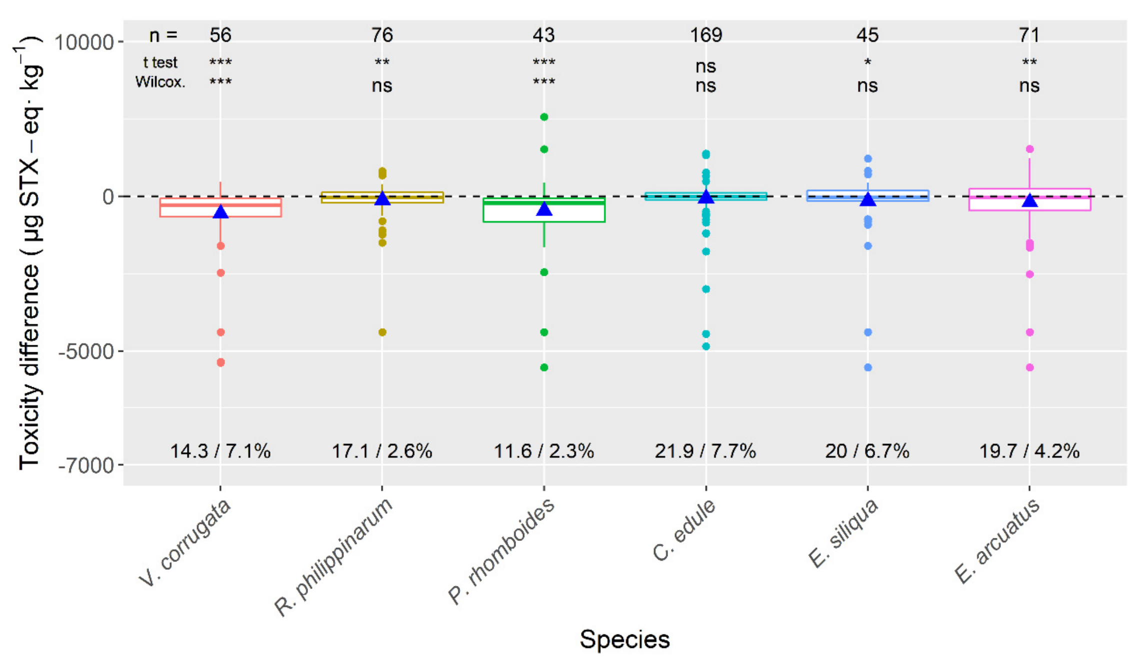

Figure S1. Differences of PSP toxicity between the main bivalves and wild mussels sampled from the same area at the same week Symbols as in Figure 3. Number in the bottom line are the percentages of samples which tested PSP positive when it was not detected in mussel (first number) or when it was detected below the regulatory level (second number). .

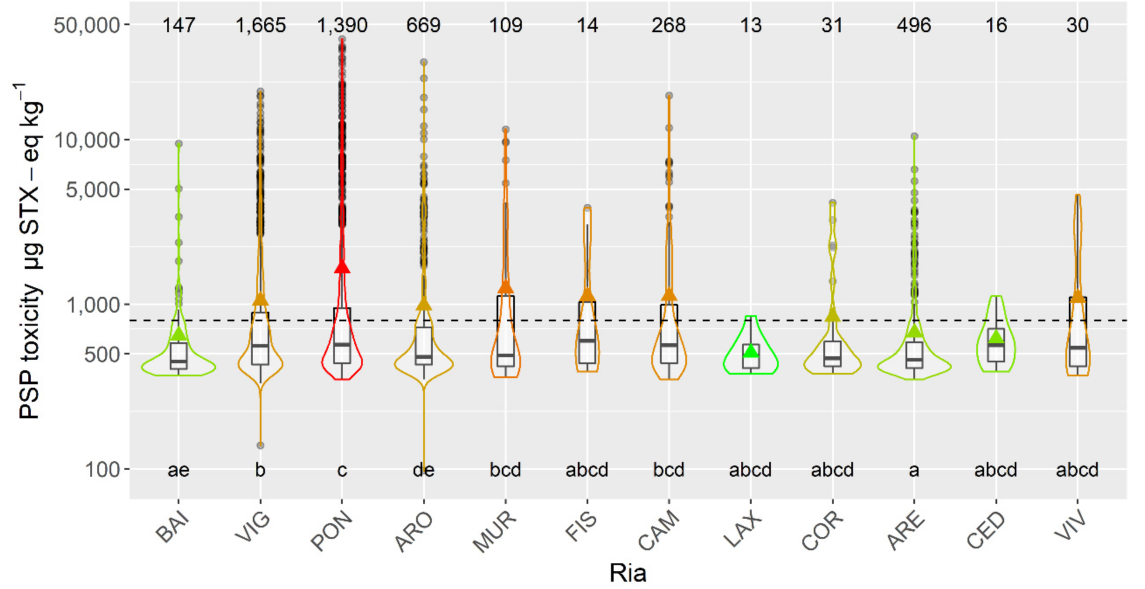

Figure S2. PSP toxicity in the main bivalve species during the toxic episodes in the Galician rías. All symbols as in Figure 3.

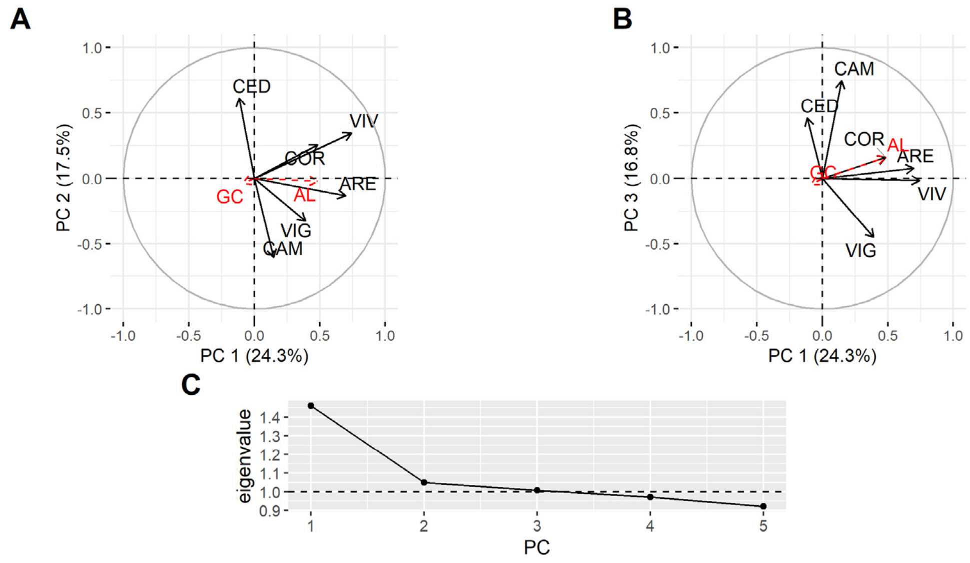

Figure S3. Loadings of the four principal components and their corresponding eigenvalues after excluding raft mussels from the analysis. AL and GC are the abundances of *Alexandrium* spp and *Gymnodinium catenatum*, and are supplementary variables which were not included in the analysis.

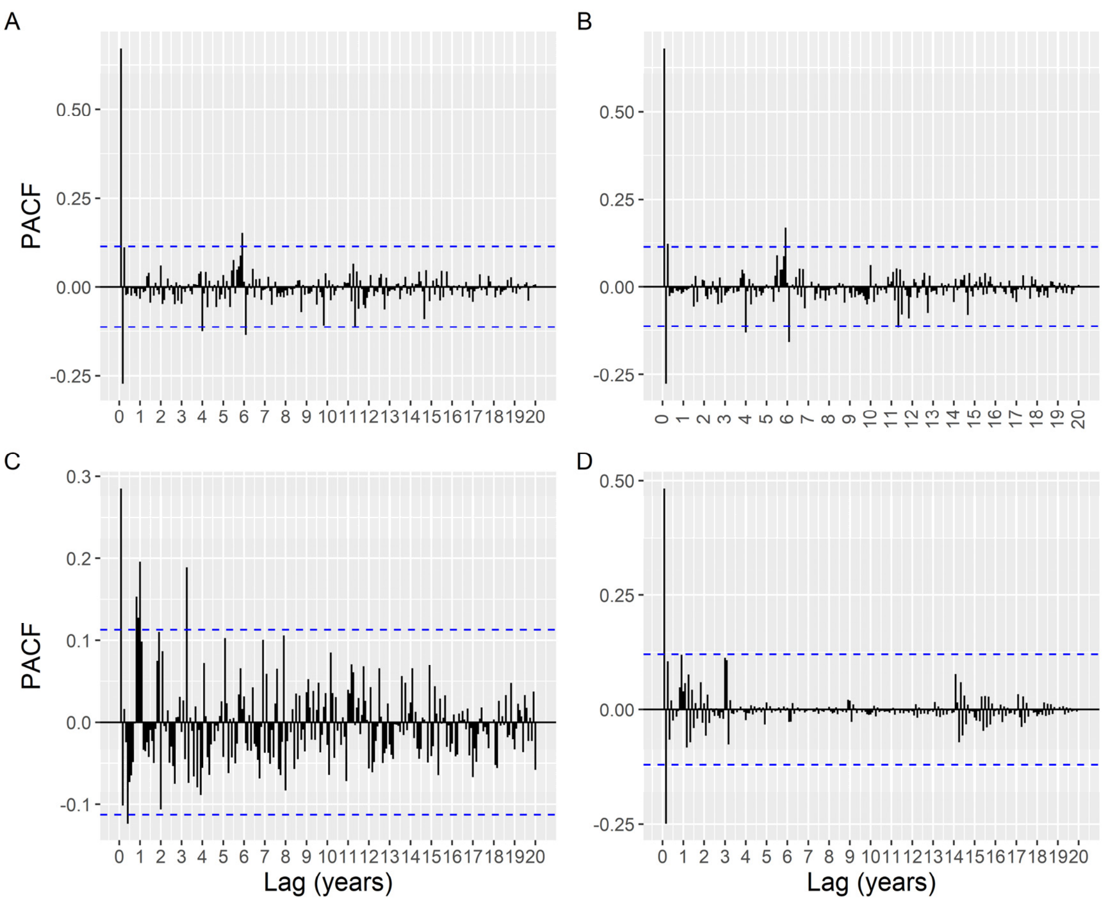

Figure S4. Partial autocorrelation plots corresponding to all Rías (A), and to the southern (B), middle (C), and northern ones (D) when all bivalve species are considered. Horizontal dashed lines are the 95% confidence limits.

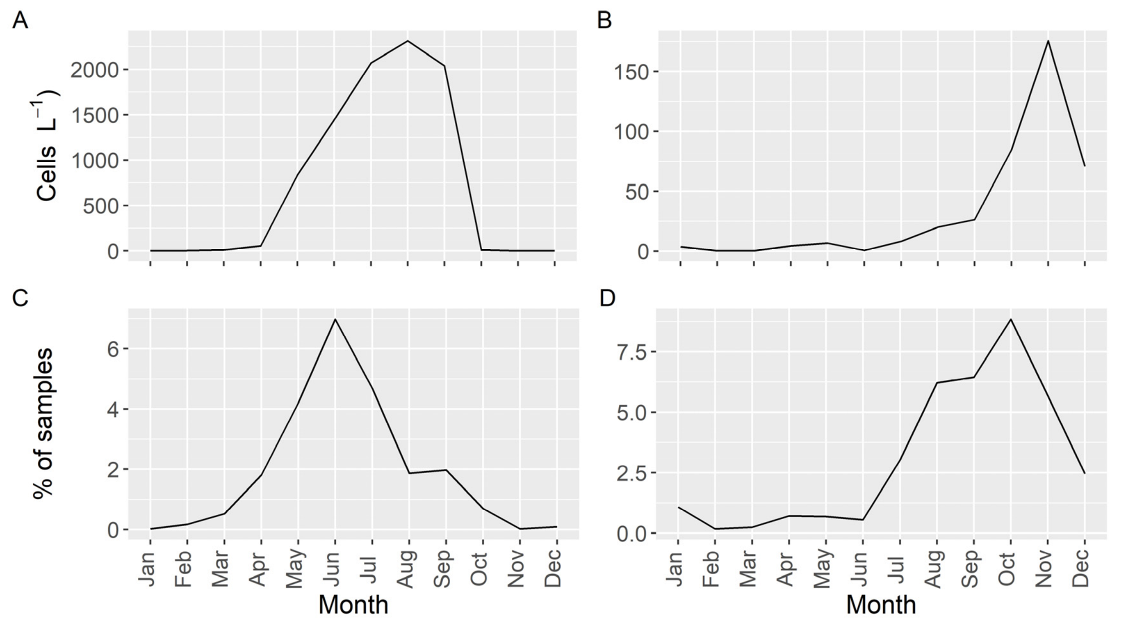

Figure S5. Average concentration per month and percentage of the samples in which the species was present for *Alexandrium* (A and C) and *Gymnodinium catenatum* (B and D).

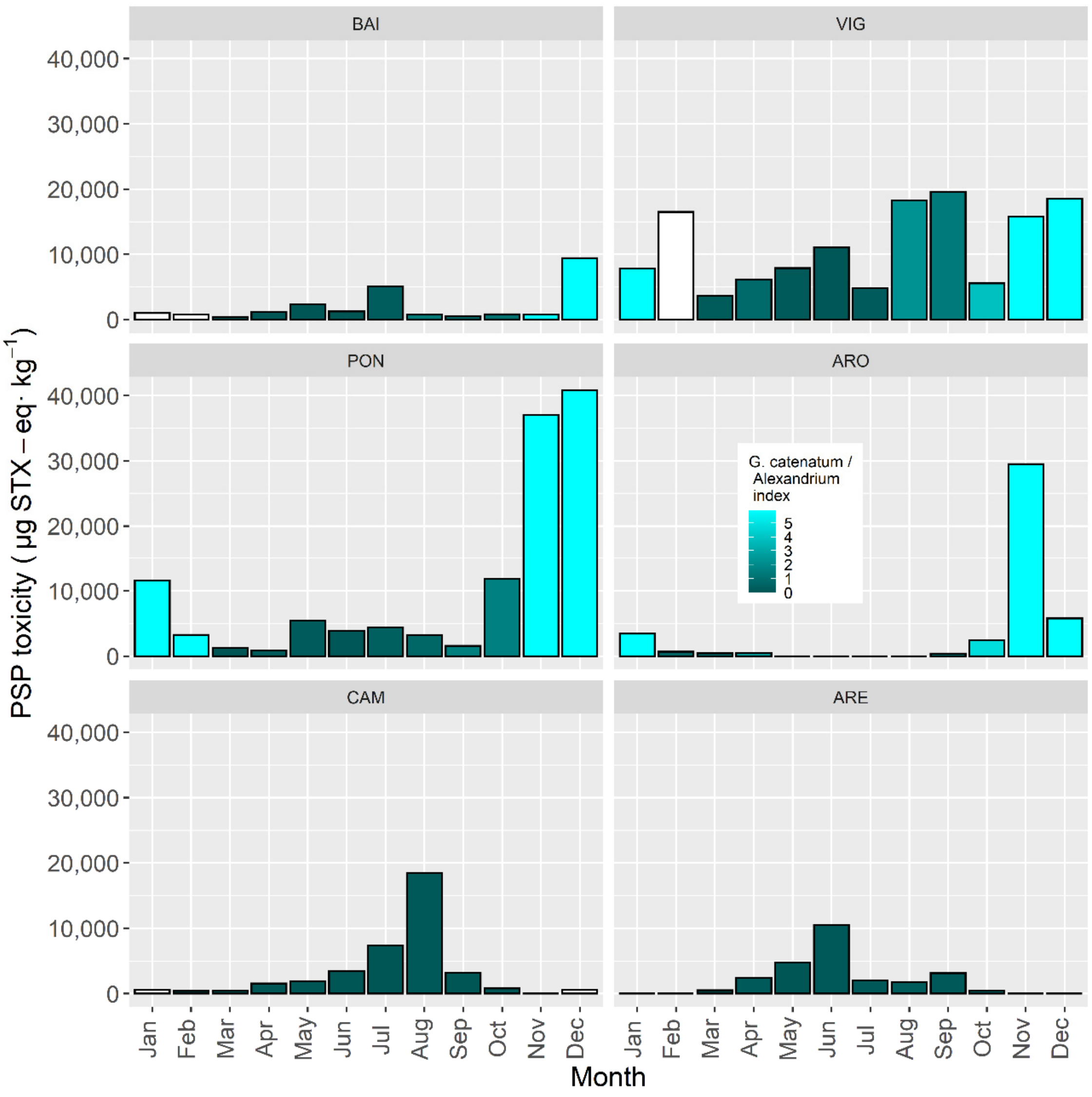

Figure S6. Maximum PSP toxicities per month in the rías with the highest PSP levels.

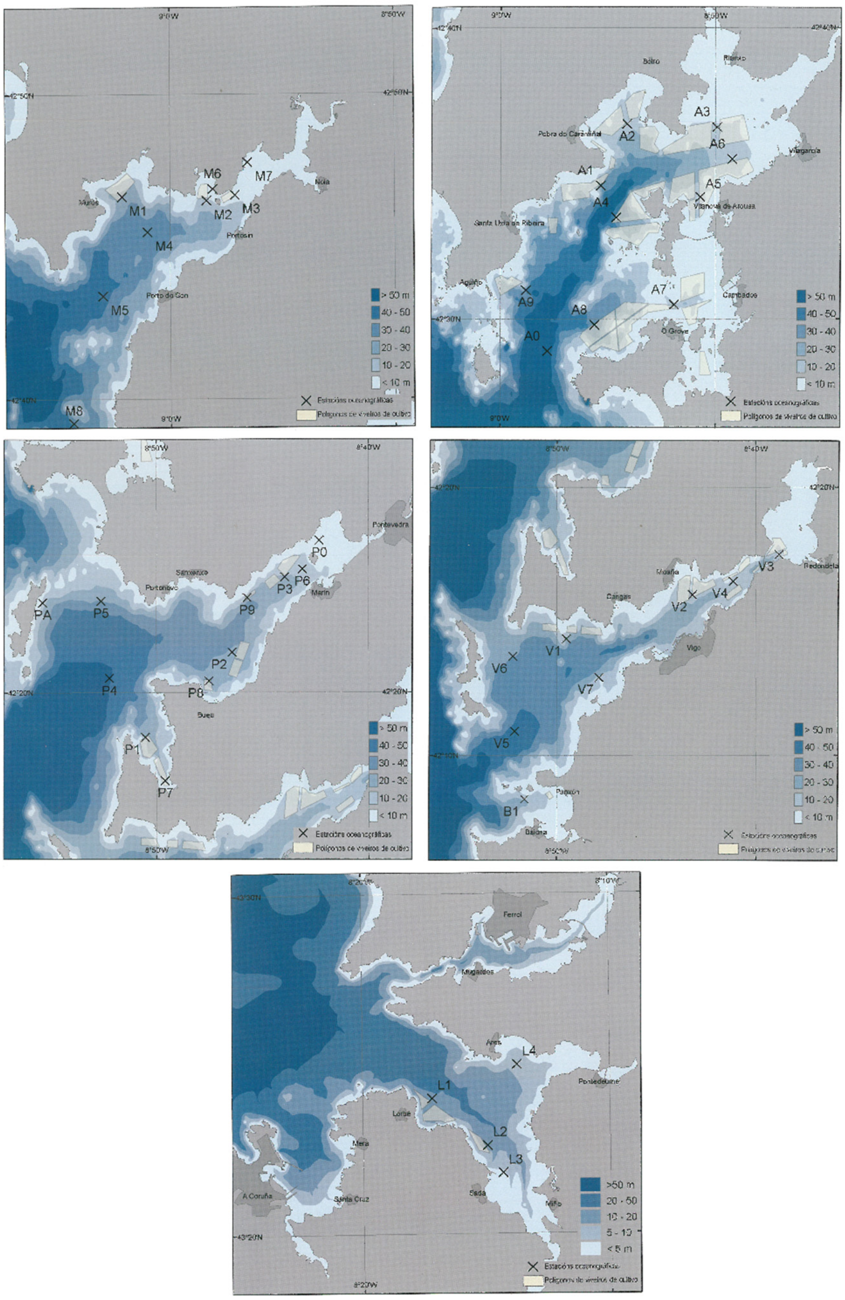

Figure S7. Phytoplankton sampling stations in the five Galician rías where raft mussels are grown.
